# Daily Intraperitoneal Administration of Rosiglitazone Does Not Improve Lung Function or Alveolarization in Preterm Rabbits Exposed to Hyperoxia

**DOI:** 10.3390/pharmaceutics14071507

**Published:** 2022-07-20

**Authors:** Giorgio Aquila, Yannick Regin, Xabier Murgia, Fabrizio Salomone, Costanza Casiraghi, Chiara Catozzi, Enrica Scalera, Matteo Storti, Francesca Stretti, Giancarlo Aquino, Giorgia Cavatorta, Roberta Volta, Carmelina Di Pasquale, Caterina Amato, Fabio Bignami, Davide Amidani, Barbara Pioselli, Elisa Sgarbi, Paolo Ronchi, Giuseppe Mazzola, Ignacio Valenzuela, Jaan Toelen

**Affiliations:** 1 Department of Preclinical Pharmacology, R&D, Chiesi Farmaceutici S.p.A., 43122 Parma, Italy; g.aquila@chiesi.com (G.A.); f.salomone@chiesi.com (F.S.); costanza.casiraghi@gmail.com (C.C.); c.catozzi@chiesi.com (C.C.); e.scalera@chiesi.com (E.S.); m.storti@chiesi.com (M.S.); c.dipasquale@chiesi.com (C.D.P.); c.amato@chiesi.com (C.A.); f.bignami@chiesi.com (F.B.); 2 Department of Development and Regeneration, Cluster Woman and Child, KU Leuven, 3000 Leuven, Belgium; yannick.regin@kuleuven.be (Y.R.); ignacio.valenzuela@kuleuven.be (I.V.); 3 Scientific Consultancy, 48640 Berango, Spain; xabi_murgia@hotmail.com; 4 Department of Veterinary Science, University of Parma, 43121 Parma, Italy; f.stretti.stage@chiesi.com; 5 Department of Pharmacokinetic, Biochemistry & Metabolism, R&D, Chiesi Farmaceutici S.p.A., 43122 Parma, Italy; g.aquino@chiesi.com (G.A.); g.cavatorta@chiesi.com (G.C.); r.volta@chiesi.com (R.V.); 6 Department of Analytic and Early Formulations, R&D, Chiesi Farmaceutici S.p.A., 43122 Parma, Italy; d.amidani@chiesi.com (D.A.); b.pioselli@chiesi.com (B.P.); e.sgarbi@chiesi.com (E.S.); 7 Department of Chemistry Research & Drug Design, R&D, Chiesi Farmaceutici S.p.A., 43122 Parma, Italy; p.ronchi@chiesi.com; 8 Portfolio Management R & D, Chiesi Farmaceutici S.p.A., 43122 Parma, Italy; g.mazzola@chiesi.com; 9 Department of Paediatrics, University Hospital Leuven, 3000 Leuven, Belgium; 10 Leuven Child and Youth Institute, KU Leuven, 3000 Leuven, Belgium

**Keywords:** thiazolidinediones, rosiglitazone, pioglitazone, preterm rabbits, bronchopulmonary dysplasia, hyperoxia, dyslipidemia, lung proteomics

## Abstract

Thiazolidinediones (TZDs) are potent PPARγ agonists that have been shown to attenuate alveolar simplification after prolonged hyperoxia in term rodent models of bronchopulmonary dysplasia. However, the pulmonary outcomes of postnatal TZDs have not been investigated in preterm animal models. Here, we first investigated the PPARγ selectivity, epithelial permeability, and lung tissue binding of three types of TZDs in vitro (rosiglitazone (RGZ), pioglitazone, and DRF-2546), followed by an in vivo study in preterm rabbits exposed to hyperoxia (95% oxygen) to investigate the pharmacokinetics and the pulmonary outcomes of daily RGZ administration. In addition, blood lipids and a comparative lung proteomics analysis were also performed on Day 7. All TZDs showed high epithelial permeability through Caco-2 monolayers and high plasma and lung tissue binding; however, RGZ showed the highest affinity for PPARγ. The pharmacokinetic profiling of RGZ (1 mg/kg) revealed an equivalent biodistribution after either intratracheal or intraperitoneal administration, with detectable levels in lungs and plasma after 24 h. However, daily RGZ doses of 1 mg/kg did not improve lung function in preterm rabbits exposed to hyperoxia, and daily 10 mg/kg doses were even associated with a significant lung function worsening, which could be partially explained by the upregulation of lung inflammation and lipid metabolism pathways revealed by the proteomic analysis. Notably, daily postnatal RGZ produced an aberrant modulation of serum lipids, particularly in rabbit pups treated with the 10 mg/kg dose. In conclusion, daily postnatal RGZ did not improve lung function and caused dyslipidemia in preterm rabbits exposed to hyperoxia.

## 1. Introduction

Bronchopulmonary dysplasia (BPD) is the major comorbidity of premature birth, affecting 35% of infants born at a gestational age < 28 weeks [1]. Extremely premature infants usually require positive pressure ventilation and supplemental oxygen in the first days of life [2], which can cause unintended harm to the immature lung. Mechanical stretch and prolonged oxygen exposure induce a pulmonary inflammatory cascade that causes lung injury by interfering with specific molecular pathways involved in the normal alveolar and microvascular development [1,3,4].

The peroxisome proliferator-activated receptor γ (PPARγ) signaling pathway plays a central role in lung development and injury/repair processes [5]. PPARγ is also involved in various lipid metabolism processes, including fatty acid catabolism, lipid trafficking, adipogenesis, and lipid storage [6]. In the lungs, PPARγ upregulation is essential for acquiring the lipogenic phenotype of alveolar fibroblasts [7]. Lipofibroblasts are located in the alveolar interstitial space and synthesize the structural proteins of the extracellular matrix, provide protection against oxidant injury, act as accessory cells (lipid source) for surfactant synthesis, and secrete growth factors that drive key downstream processes for normal alveolar and vascular differentiation [5,8].

BPD triggers, including excessive mechanical stretch and hyperoxia, downregulate PPARγ signaling and upregulate the transforming growth factor (TGF)-β/Wnt pathway, which promotes excessive mesenchymal transdifferentiation into the myofibroblast phenotype, eventually leading to an abnormal lung injury/repair process characterized by reduced alveolarization and impaired angiogenesis [9,10,11,12]. In light of these findings, PPARγ signaling activation by specific synthetic agonists has been proposed as a potential treatment of BPD [13].

Thiazolidinediones (TZD), such as rosiglitazone (RGZ) and pioglitazone (PGZ), are a class of synthetic antidiabetic drugs that act as potent PPARγ agonists, modulating the expression of several genes involved in glucose and lipid metabolism [14]. Given their role in the activation of the PPARγ pathway, TZDs have also been investigated in the context of BPD, achieving promising results in experimental models. For instance, antenatal TZDs induce PPARγ expression, accelerate lung maturation (e.g., increased surfactant proteins (SP) expression) and prevent the morphological and molecular changes induced by hyperoxia [15,16,17,18]. Similarly, postnatal TZDs delivered to newborn rodent BPD models promote lung maturation (SP expression and lamellar body count), increase PPARγ and vascular endothelial growth factor (VEGF) expression, preserve the lipofibroblast/myofibroblast ratio, and attenuate the alveolar simplification observed after hyperoxia exposure [10,16,19,20,21,22,23,24]. Although previous investigations in newborn rodent models have set the proof-of-concept for the use of postnatal TZDs as potential BPD treatment, the efficacy of postnatal TZDs has not been investigated yet in a preterm animal model of BPD. 

Preterm rabbit pups exposed to hyperoxia represent an alternative model for the early efficacy testing of pharmacological interventions for BPD treatment. Unlike rodents, which display fully functional lungs at birth, preterm rabbits delivered at 28 days of gestation (term 31 days) develop mild-to-moderate respiratory distress [25,26,27]. Moreover, although both rodents and rabbits display the BPD phenotype after exposure to hyperoxia, changes in lung morphology have been described after just 24 h of hyperoxia in rats [24]. In contrast, at least 5 days of hyperoxia (95% O_2_) are required to induce significant parenchymal and vascular changes in premature rabbits [28]. Since a differential response to injury may also be reflected by a different response to TZDs across species, the present study was designed to investigate the efficacy of the daily, postnatal administration of TZDs in preterm rabbits exposed to hyperoxia. For that purpose, we first investigated the PPARγ receptor selectivity of three TZDs, namely, RGZ, PGZ, and DRF-2546, their plasma and tissue binding, and their in vitro epithelial permeability through Caco-2 monolayers. In the second part of the study, the pharmacokinetics of RGZ after either local (intratracheal administration) or systemic delivery (intraperitoneal injection) were investigated in premature rabbits. After that, the effects of daily RGZ administration over respiratory functional parameters and alveolar morphometry were evaluated in preterm rabbits exposed to hyperoxia (95% O_2_) for 7 days. In addition, a biochemical assessment of blood lipids and a comparative lung proteomics analysis were performed to respectively quantify the levels of circulating lipids and investigate the differential protein expression induced by RZG in preterm rabbits exposed to hyperoxia. 

## 2. Materials and Methods

### 2.1. In Vitro Selectivity Assay for Peroxisome Proliferator-Activated Receptor of Thiazolidinediones

A human PPARγ nuclear reporter assay was purchased from Indigo Biosciences Inc. (Cat. #IB00101, State College, PA, USA) and was performed according to the manufacturer’s instructions. Briefly, the reporter cells of the assays include a luciferase reporter gene functionally linked to a PPARγ-responsive promoter. Thus, quantifying changes in luciferase expression in the treated reporter cells provides a sensitive surrogate measure of the changes in PPARγ activity. Rosiglitazone standard (Indigo Biosciences, Inc.) was used as reference agonists for PPARγ. Reporter cells were incubated for 24 h at 37 °C in a humified incubator at 5% CO_2_ with three different TZDs—RGZ, PGZ, or DRF-2546 [13]—at a maximum concentration of 10 μM, with seven serial dilutions, and a final DMSO concentration of 0.03% v/v. After incubating with each TZD, luminescence was quantified using a plate-reading luminometer (GloMax-Multi+, Promega Corporation, Madison, WI, USA). A sigmoidal dose–response analysis was performed to determine the half-maximal effective concentration (EC50) values for each TZD (GraphPad Prism 8.4.3, San Diego, CA, USA). 

### 2.2. Plasma Protein Binding, Lung Tissue Binding, and Caco-2 Permeability Assay

#### 2.2.1. Plasma Protein Binding

In vitro plasma protein binding was determined in preterm rabbit plasma (New Zealand White Pooled Gender, BIOIVT, Hicksville, NY, USA) by equilibrium dialysis using Thermo Fisher Scientific Pierce RED rapid equilibrium dialysis devices (Device Inserts 8K MWCO, Thermo Fisher Scientific, Waltham, MA, USA). The assay was performed using a Tecan Evoware robotic system (Männedorf, Switzerland). Test compounds and the positive control were diluted in the pooled plasma (three donors) to a final concentration of 5 mM and then dispensed into the red chamber of the RED device inserts. Phosphate-Buffered Saline (PBS, from Merck KGaA, Darmstadt, Germany) was dispensed into the other side. Upon completion of 4 h of incubation (37 °C under shaking), equal aliquots were removed from each chamber and matrix matched with even volumes of plasma or buffer for LC-MS/MS analyses.

*Free* and *Bound* percentages of the compounds were calculated as follows:

(1) $$\%Free=\frac{Area\;buffer\;sample}{Area\;plasma\;sample}\times 100$$

(2) % Bound=100−%Free

#### 2.2.2. Lung Tissue Binding

In vitro lung tissue binding was also determined by equilibrium dialysis as described above. Preterm rabbit lungs (New Zealand White, BIOIVT) were homogenized using a Precellys tissue homogenizer (Bertin instruments, Montigny-le-Bretonneux, France). Precellys lysing vials (CK28-R, Bertin instruments) were filled with lung and buffer according to the following proportion: 1 g of tissue and 5 mL of ice-cold PBS (dilution 1:6). At the end of the homogenization process, a pool of lung homogenate was prepared mixing equal amount of homogenate from three different donors. Test compounds and the positive control were diluted in the pooled lung homogenate to a final concentration of 5 mM and then dispensed into the red chamber of the RED device inserts. PBS was dispensed into the other side. Upon completion of 4 h of incubation (37 °C under shaking), equal aliquots were removed from each chamber and matrix matched with even volumes of lung homogenate or buffer for LC-MS/MS analyses.

*Free* and *Bound* percentages of the compounds were calculated as follows:

(3) $$\%Free=\frac{Area\;buffer\;sample}{Area\;lung\;homogenate\;sample}\times 100$$

(4) % Bound=100−%Free

#### 2.2.3. Caco-2 Permeability Assay

Caco-2 cells were purchased from ReadyCell (Barcelona, Spain). The cells arrived already seeded and differentiated (21-days) in 96-well plates (CacoReady 96-well kits, ReadyCell). Cells were maintained in a cell culture medium: Dulbecco’s modified Eagle’s medium (DMEM) 1 g/L glucose containing 10% fetal bovine serum, 1% L-glutamine 200 mM, and 1% penicillin (10,000 U/mL)–streptomycin (10 mg/mL), all from Merck KGaA. The buffer used in the assay was Hanks Balanced Salt Solution (HBSS, Merck). Control compounds were: sulpiride (low permeability control), metoprolol (high permeability control), talinolol (P-glycoprotein transport (*P_gp_*) substrate control), and GF120918 (Elacridar, *P_gp_* inhibitor), all purchased from Merck KGaA.

The day of the experiment, the integrity of the cell monolayers was assessed before and after the assay by measuring the transepithelial electrical resistance (TEER). The permeability of the compounds across the cell monolayer was determined by measuring their transport in both directions: apical to basolateral (A → B) and basolateral to apical (B → A) in the absence and presence of Elacridar (a known P-gp inhibitor). The donor working solution was prepared by dilution of the DMSO stock of the test compound or positive control with HBSS (pH 7.4) to 10 µM. For A → B directional transport, the donor working solution (with test compound or positive control, with or without P-gp inhibitor) was added to the apical (A) and HBSS was added to the basolateral (B) compartment. For B → A directional transport, the donor working solution (with positive control or test compound 10 mM, with or without P-gp inhibitor) was added to the basolateral (B) and HBSS was added to the apical (A) compartment. The assay was performed with a Hamilton Star Lab robotic system. Samples were collected at *t*_0_ and 2 h after incubation (5% CO_2_, 95% relative humidity, 37 °C) for LC-MS/MS analyses.

Apparent permeability (*P_app_*) was calculated with the following equation:

(5) $$P_{app}\left(\frac{\mathrm{cm}}{\sec}\right)=\left(\frac{Vr}{C_{0}}\right)\times \left(\frac{1}{A}\right)\times \left(\frac{Cr\left(t\right)}{t}\right)$$

where *Vr* is the volume of the solution in the receiving compartment (mL); *C*_0_ is the initial test compound concentration in the donor compartment (expressed as area ratio); A is the membrane surface area (0.14 cm^2^); *Cr* (t) is the measured concentration of the receiver well at time *t*_120_ (expressed as area ratio); and t is time of incubation (sec).

The efflux ratio (*ER*) was also measured to understand if the compounds undergo active efflux:

*Efflux ratio* = *P_app_* [B > A]/*P_app_* [A > B] (6)

(7) $$ER\;Inhibition\;\%=100-\left(\frac{Efflux\;ratio\;with\;inhibitor}{Efflux\;ratio\;without\;inhibitor}\right)\times 100\;$$

#### 2.2.4. Liquid Chromatography–Tandem Mass Spectrometry (LC/MS/MS) Method

An Agilent 1200 liquid chromatography system was used (Agilent, Palo Alto, CA, USA). The auto-sampler and column oven were kept at 10 °C and 30 °C, respectively. Chromatographic separation was achieved on a Phenomenex EVO C18, 50 × 2.1 mm, 2.6 u (Phenomenex, Torrance, CA, USA) using 0.1% formic acid in water (Carlo Erba reagents S.A.S., Val De Reuil Cedex, France; Solvent (A) and 0.1% formic acid in acetonitrile (Carlo Erba reagents S.A.S.; solvent (B) as mobile phases. The gradient program for PGZ and DRF-2546 was 0.0–0.5 min, 10% B; 0.5–3.5 min, gradient to 90% B; 3.5–5 min, 90% B; 5–6 min, gradient to 10% B; 6.0–10.0 min, 10% B. For RGZ: 0.0–0.5 min, 5%B; 0.5–3.5 min, gradient to 90% B; 3.5–5 min 90% B; 5–6 min gradient to 5% B; 6–10 min 5% B.

The flow rate and injection volume were set at 0.4 mL/min and 2.5 μL for PGZ and DRF-2546. For RGZ, the flow rate and injection volume were set at 0.3 mL/min and 5 μL. The HPLC system was coupled to an AB SCIEX 4500 Q-TRAP triple quadrupole mass spectrometer (AB Sciex, Foster City, CA, USA) equipped with an ESI source. The mass spectrometer operated in positive ion mode. Ion spray voltage was set at 5000 V (PGZ and DRF-2546) and 5500 V (RGZ) while the temperature was set at 500 °C (PGZ and DRF-2546) and 450 °C (RGZ). For the PGZ and DRF-2546 curtain gas, source gas 1 and source gas 2 were set at 20, 40, and 35, respectively, while for RGZ they were set at 20, 40, and 30, respectively. Quantification was operated in multiple reaction monitoring (MRM) mode using the following *m*/*z* transitions: 357.13→134.00 for PGZ, 410.097→187.100 for DRF-2546, and 358.15→135.200 for RGZ.

### 2.3. Animal Care and Cesarean Section

For the pharmacokinetic profiling study, pregnant New Zealand White rabbits were provided by Charles River (Domaine des Oncins, France) and the experiments were approved by the internal AWB (Animal Welfare Body) at Chiesi Farmaceutici and the Italian Ministry of Health (Protocol No. 899/2018-PR). For efficacy studies, time-mated pregnant hybrid rabbits (New Zealand x Flemish Giant) were provided by the animal facility of KU Leuven and the experiments were approved by the Ethics Committee for Animal Experimentation of the Faculty of Medicine of KU Leuven. All animal experiments were performed in compliance with the European guidelines on animal welfare (P165/2021).

In all experiments, preterm rabbit pups were extracted by caesarean section at 28 days of gestational age (GA; term 31 days), as previously described [26]. Briefly, does were pre-medicated with intramuscular ketamine (35 mg/kg; Nimatek^®^, Eurovet Animal Health BV, Bladel, The Netherlands) and xylazine (6 mg/kg; XYL-M^®^; VMD, Arendonk, Belgium), placed in the supine position, and euthanized with an intravenous bolus of 1 mL T61 (Intervet Belgium, Mechelen, Belgium), which contains a mixture of 200 mg of embutramide, 50 mg of mebezonium, and 5 mg of tetracaine hydrochloride. Exceptionally, does were euthanized with a 100 mg/kg overdose of pentothal sodium (MSD Animal Health, US) in the pharmacokinetic studies. The abdomen was opened through a low midline abdominal incision and the pups were rapidly extracted through hysterotomy. At delivery, pups were dried, stimulated, and placed on soft bedding inside an incubator under controlled conditions of hyperoxia (95% O_2_), temperature (32 °C), and humidity (50–60%).

### 2.4. Pharmacokinetic Profiling of Rosiglitazone

#### 2.4.1. In Vivo Phase

A total of 30 preterm rabbits with a GA of 28 days were featured in the pharmacokinetic study. After delivery, all pups were put in hyperoxia (95% oxygen) for 1 h. Surviving pups were randomized to either receive an intratracheal (IT, local delivery, n = 15) or intraperitoneal (IP, systemic delivery, n = 15) dose of 1 mg/kg of RGZ. For IP injections, RGZ (10 mg/mL in DMSO) was dissolved in H_2_O + 10% Tween-80 at 0.4 mg/mL and then injected in the right flank based on the pups’ body weight. For IT injections, RGZ (50 mg/mL in DMSO; Merck KGaA) was dissolved in 80 mg/mL of Poractant alfa (Curosurf, Chiesi Farmaceutici, Parma, Italy) at 0.4 mg/mL; the injection volume was based on the pups’ body weight (2.5 mL/kg), as previously described [29].

Terminal blood and lung samples were obtained immediately after delivery (*t*_0_), and then at 15 min, 1 h, 6 h, and 24 h (n = 3 samples per time point) following IT or IP RGZ injections. Before sample collection, all pups were euthanized with a pentothal sodium overdose. All samples were stored at −20 °C until analysis.

#### 2.4.2. Bioanalysis and Pharmacokinetic Analysis

Bioanalysis and quantification were performed using a fit-for-purpose LC/MS/MS method developed internally (LC and MS conditions already described in Section 2.2). Plasma and lung homogenate samples (lungs were homogenized with a water/acetonitrile 50/50 mixture) were extracted by means of protein precipitation with 3 volumes of acetonitrile (Sigma-Aldrich Co, St. Louise, MO, USA), and the supernatant was directly injected into the LC/MS/MS system.

The plasma and lung concentrations of RGZ were determined using a calibration curve prepared in the corresponding blank rabbit matrix (range of calibration: 2–500 ng/mL for plasma; 12–6000 ng/g for lungs) and the accuracy of the quantitation was verified using quality controls prepared in the same matrix and back-calculated on the curve. Accuracy of the back-calculated analyte concentration was within ± 15% of the calibration standard nominal value (except 20% for the lower limit of quantitation). Values falling outside these limits were discarded, but at least 75% or a minimum of 6 calibration standards were used for the curve. At least 67% of the quality control samples showed accuracy within ±15% of the nominal value and there was at least one valid quality control sample for each concentration level.

Pharmacokinetic parameters were calculated by non-compartmental analysis using Phoenix WinNonlin (Certara L.P., Princeton, NJ, USA). The following PK parameters were calculated: T_max_, time of the maximum concentration; C_max_, maximum concentration; t_1/2_, half-life (calculable only in case of well-defined elimination phase); AUC_last_, area under the curve up to the last time point.

### 2.5. Pulmonary Efficacy Studies: Daily, Intraperitoneal Rosiglitazone Administration at 1 mg/kg

A total of 31 preterm rabbits with a GA of 28 days delivered from 4 does were included in the efficacy study. After delivery, newborn pups were put in hyperoxia (95% oxygen) for 1 h. Surviving pups were exposed to hyperoxia (95% O_2_) for 7 days and randomized to receiving daily (from postnatal day (PND) 0 to PND 6) intraperitoneal injections of RGZ at 1 mg/kg (n = 15) or the vehicle (n = 16).

Pups were placed on a sterilized soft bedding and remained in the incubator except for the feeding and treatments. Pups were fed 4 h after delivery on PND 0 with a milk replacer (Day One^®^, Protein 30%, Fat 50%; FoxValley, Aurora, IL, USA). Probiotics, electrolytes, and vitamins are added during the first 7 days (Bio-Lapis^®^; Probiotics International Ltd., Somerser, UK) and immunoglobulins during the first 2 days (Col-o-Cat^®^, SanoBest, Hertogenbosch, Netherlands). The standard feeding protocol consisted of incrementally increasing the amounts of milk replacer (PND 0: 40 mg/kg, PND 1: 50 mg/kg/day, PND 2: 75 mg/kg, PND 3–6: 100 mg/kg/day). On Day 2, vitamin K1 was administered intramuscularly (0.002 mg/kg BW, Konakion pediatrique^®^; Roche, Basel, Switzerland).

#### 2.5.1. Lung Function Testing

At Day 7, invasive lung function testing was performed using the forced oscillation technique with the flexiVent^TM^ apparatus (SCIREQ, Montréal, Canada). Pups were anesthetized with intramuscular ketamine (35 mg/kg) and xylazine (6 mg/kg) before a tracheostomy was performed, and an 18-gauge metal needle was inserted in the trachea and tied. Pups were connected to the volume-controlled mechanical ventilation of the flexiVent^TM^ (SCIREQ, tidal volume 10 mL/kg, frequency of 120 breaths/min, PEEP of 3 cmH_2_O). All pups were euthanized with a pentothal sodium overdose.

The following parameters were measured: total inspiratory capacity, resistance of the respiratory system (R_rs_), lung tissue damping (G), lung tissue elastance (H), and static compliance (C_st_). Three consistent measurements were obtained, with a coefficient of determination >0.95 as the limit to accept the measurement. The average of three measurements was calculated and used in the analysis.

#### 2.5.2. Radial Alveolar Count

At necropsy, lungs were removed en bloc and a 20-gauge catheter was secured inside the trachea. Lungs were fixed with 10% buffered formalin (Sigma-Aldrich) for at least 4 h under constant pressure (25 cmH_2_O) using a custom-made fixation device to ensure homogenous fixation pressures. After removal from pressure fixation, the lungs were first left in formalin for at least 24 h and then transferred to 70% ethanol.

The left lung and the right lungs were embedded in paraffin, cut, and stained with hematoxylin and eosin (H&E) following standard histology protocols. The radial alveolar count (RAC) was performed by an expert pathologist blinded to the experimental design by drawing a perpendicular line from the lumen of the terminal bronchiole to the nearest connective tissue septum or pleural margin and the number of saccules or alveoli crossed by this line was counted as previously described [30]. In total, 20 measurements were performed for each lung pair.

### 2.6. Daily, Intraperitoneal Rosiglitazone Administration at 10 mg/kg

An independent experimental session investigated the efficacy of RGZ at a higher dose (10 mg/kg). In total, 36 preterm rabbits with a GA of 28 days delivered from 4 does were randomized to either the control group (n = 18) or the RGZ 10 mg/kg group (n = 18 per group). Hyperoxia exposure, RGZ administration, animal care and feeding procedures, lung function testing, RAC, and sample collection were performed as described above.

### 2.7. Quantification of Blood Lipid Levels

Whole blood samples from pups in the RGZ (1 and 10 mg/kg groups) and control groups (n = 6 per group) were obtained though cardiac puncture. Briefly, a 22G needle was inserted into the left ventricle and approximately 1.5 mL of whole blood were collected in K2-EDTA tubes and stored on ice. Within 60 min after blood draw, samples were centrifuged at 1500× *g* for 15 min and the serum was immediately stored at −80 °C. At the day of quantification, samples were gently thawed overnight at 4 °C and the level of triglycerides (TG), total cholesterol, high-density cholesterol (HDL), and non-HDL were determined using a Cobas 8000 modular analyzer (Roche).

### 2.8. Quantitative Proteomic Analysis by Mass Spectrometry

Approximately one quarter of the right lung from pups in the RGZ (1 and 10 mg/kg groups) and control groups (n = 6 per group) were used for the proteomic analysis. Lung samples were resuspended in 5 mL of PBS and homogenized using a gentleMACS^TM^ Dissociator (Miltenyi Biotec Inc. Bergisch Gladbach, Germany). Three consecutive freezing (by immersion in liquid nitrogen) and thawing cycles were carried out on lung homogenates to further promote cell lysis. Cellular debris and insoluble material were pelleted by centrifugation (10,000× *g*, at 4 °C for 5 min), and the supernatant was collected for protein quantification by a Bradford assay.

In total, 50 μg of proteins from each lung homogenate were resuspended in a final equal volume of 50 μL using ammonium bicarbonate (pH 8.0), containing 3.5% sodium dodecyl sulfate (SDS), and finally processed using S-Trap micro columns (ProtiFi, Huntington, PA, USA). Protein samples were exposed for 5 min at 95 °C for thermal denaturation, and then reduced and alkylated with dithiothreitol and iodoacetamide, respectively. Protein denaturation was completed by addition of phosphoric acid to a final concentration of 1.2% *w*/*v* and the volume was increased 6-fold with 100 mM TEAB in 90% methanol. The samples were then loaded on S-Trap micro columns and washed 3 times with 150 μL of 100 mM TEAB in 90% methanol. Proteins were trapped in the column resin for 16 h incubated at 37 °C with 20 μL of 50 mM TEAB containing 3.3 μg of trypsin (Trypsin Gold, Promega Corporation). After digestion, the peptides were eluted using 40 μL of 50 mM TEAB, 0.2% formic acid, and 0.2% formic acid in 50% acetonitrile, and collected in the same tube. Peptide volume was dried under vacuum and resuspended in 50 μL of 0.1% trifluoroacetic acid in water. An aliquot of 1 μg of peptides was separated by a Dionex UltiMate3000 nanoUHPLC system equipped with a PepMap RSLC C18 column (Thermo Fisher Scientific) at 35 °C using a four steps gradient at a flow of 0.3 μL/min (80% acetonitrile in 0.1% formic acid from 5% to 29% in 203 min; from 29% to 50% in 44 min; from 50% to 99% in 16 min; and 36 min of column equilibration).

Peptide fractions were analyzed by a Fusion Lumos mass spectrometer (Thermo Fisher Scientific) operating in data-dependent acquisition (DDA) mode, with the most abundant ions selected for fragmentation by collision-induced dissociation (CID). Protein identification and data analysis were performed using Proteome Discoverer 2.4 (Thermo Fisher Scientific) and Perseus software [31], respectively. Proteins were included in the proteomic analysis in case of (i) high confidence identification; and (ii) at least 2 peptides identified as “unique” for a protein. Protein abundances were deemed as significant based on a cut-off of *p*-value < 0.05 coupled to a differential expression of 1.5-fold (i.e., log 2-fold-change = 0.5849) comparing the RGZ administered animal groups and untreated control group. Significantly upregulated and downregulated proteins were employed for the functional enrichment analysis and generation of a physiological pathways map using the web-based portal Metascape 3.5 [32]. The enrichment process was carried out setting the values of 3, 0.01, and 1.5 for the parameters Min. Overlap, *p*-value Cutoff, and Min. Enrichment, respectively. In this paper, we explain the most significant pathways belonging to the groups generated by the Metascape enrichment analysis and with a Log *q*-value < −4.5.

### 2.9. Statistical Analysis

For efficacy outcomes (RAC and lung function) and blood lipid quantification, data sets were analyzed for normality using the Shapiro–Wilk test. Data fitting the criteria of a normal distribution were analyzed using an unpaired, two-sided t-test. Non-parametric data were analyzed using the Mann–Whitney test. GraphPad Prism software (GraphPad Prism 8.4.3, San Diego, CA, USA) was used for statistical analysis. Unless otherwise stated, all data are presented as the mean ± standard deviation and differences with *p*-values < 0.05 were considered significant.

## 3. Results and Discussion

Despite advances in neonatal care such as antenatal steroids, non-invasive ventilation techniques, and closely targeted supplemental oxygen levels, BPD remains the major comorbidity associated with premature birth [1,2]. To date, just a few pharmacological interventions are available in the context of BPD (i.e., caffeine and postnatal glucocorticoids) [1], while the development of novel pharmacological treatments remains an unmet clinical need.

PPARγ signaling is downregulated by the main triggers of BPD [9,10], and is associated with the activation of abnormal lung/injury repair mechanisms that result in hypoalveolarization and abnormal vascular development. Notably, synthetic TZDs induce PPARγ expression and signaling, and have been shown to counteract the deleterious effects caused by hyperoxia in the lungs of newborn rodents [13]. Here, we performed the in vitro characterization of three types of TZDs and selected RGZ to assess its pharmacokinetics and in vivo efficacy of daily postnatal administration to preterm rabbits exposed to hyperoxia.

### 3.1. RGZ Has the Highest Affinity for Human PPARγ among TZDs

We first investigated the selectivity of three TZDs (RSZ, PGZ, and DRF-2546) for the human PPARγ. RGZ and PGZ have been long used as antidiabetic drugs [33], and have been explored in rodent models as experimental treatments for BPD [10,21,22,24]. DRF-2546 (also known as PHT-46) is another synthetic TZD that reached clinical development for the treatment of chronic obstructive pulmonary disease as an inhaled formulation [34]. Interestingly, DRF-2546 showed superior plasma glucose and TG reductions compared with RGZ and PGZ in the db/db diabetic mice [35,36].

**Figure 1:**
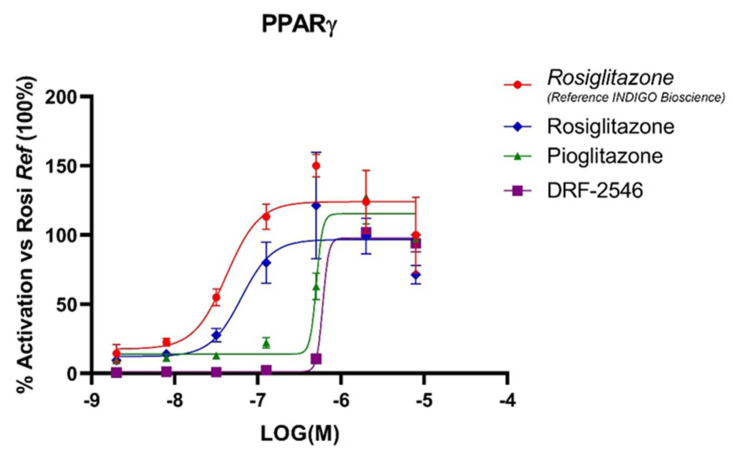
In vitro selectivity of different thiazolidinediones for the human peroxisome proliferator-activated receptor γ (PPARγ). Results are shown as the percentage activation of each thiazolidinedione with respect to the reference compound (rosiglitazone from Indigo Biosciences).

The assay demonstrated a higher selectivity of RGZ for human PPARγ (EC50 = 63 nM), showing, respectively, an 8- and 9-fold higher affinity for the receptor compared with PGZ (EC50 = 500 nM) and DRF-2546 (EC50 = 602 nM, Figure 1). Higher selectivity of RGZ for PPARγ compared with PGZ has already been described and is reflected by the difference in the maximum recommended dose for each drug in the management of type 2 diabetes (RGZ 8 mg/day vs. PGZ 45 mg/day), which is 7.5-fold lower for RGZ [14].

### 3.2. TZDs Show High Epithelial Permeability and Plasma Protein and Lung Tissue Binding

The pharmacokinetic profile of TZDs has not been investigated in animal models of BPD. Experimental protocols in neonatal rodents have mainly reported RGZ delivery via intraperitoneal injection [10,20,23,24]. Nevertheless, oral [22] and aerosol administration [21] of RZG have been reported. Takeda et al. impregnated the chow of nursing mice with 2.5–10 mg/kg of RGZ and verified that RGZ crossed into the mother’s milk and then reached the mice pups’ lungs, detecting 90.7 pg/g of RGZ in the lung tissue of PND 13 mice [22]. Morales et al. delivered a nominal dose of 3 mg/kg of nebulized RGZ to newborn rat pups confined in an inhalation chamber over 30 min and reported plasma levels of RGZ as high as 0.4 mg/mL 24 h following nebulization [21].

Moreover, RGZ is also able to cross the placental barrier since plasma levels of 0.35 μg/mL were reported by Rehan et al. in newborn mice whose mothers received two antenatal doses of 3 mg/kg of RGZ 48 and 72 h prior to delivery [16].

These studies indicate a high permeability of RGZ through biological membranes, which was confirmed by our in vitro assay performed in Caco-2 cell monolayers. The assay showed high bidirectional (A–B and B–A) *P_app_* values for all TZDs, including RGZ (Table 1). Moreover, the epithelial permeability was independent of P_gp_-mediated transport with no involvement of efflux transporters. All three TZDs displayed high plasma and lung tissue binding.

Based on the similar epithelial permeability and the higher selectivity for PPARγ, RGZ was selected for pharmacokinetic and efficacy studies.

### 3.3. Rosiglitazone Displays a High Blood-to-Lung Delivery Irrespective of the Administration Route

**Figure 2:**
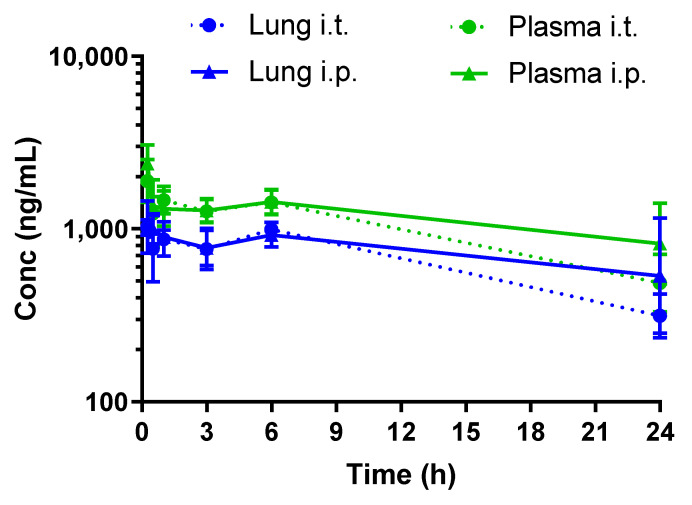
Pharmacokinetic profiles of rosiglitazone (1 mg/kg) following intratracheal (i.t.) or intraperitoneal (i.p.) administration to preterm rabbits.

We investigated the pharmacokinetics of RGZ either delivered intratracheally or via intraperitoneal injection. In our preterm rabbit model, RGZ peaked at 0.25 h in plasma and lung tissue, irrespective of the administration method, showing detectable levels in both compartments up to 24 h (Figure 2 and Table 2).

The pharmacokinetic profiling of RGZ demonstrated a rapid equilibrium between lung and plasma after both intratracheal and intraperitoneal administration, in agreement with the observed in vitro epithelial permeability in Caco-2 cells. Intraperitoneal administration rapidly exposed the pups’ lungs to RGZ, with levels above 0.5 μg/mL in both plasma and lung 24 h after exposure. Of note, plasma levels of 0.35 μg/mL were reported by Rehan et al. to protect the lungs of newborn rats from hyperoxia [24]. Intratracheal administration did not improve the lung-to-plasma partition, denoted by the AUC_Lung_/AUC_Plasma_ ratio, which remained the same irrespective of the delivery method. Therefore, the intraperitoneal administration route was selected for the in vivo efficacy studies.

### 3.4. Daily Intraperitoneal Administration of Rosiglitazone Does Not Improve Lung Function or Alveolarization in Preterm Rabbits Exposed to Hyperoxia

**Figure 3:**
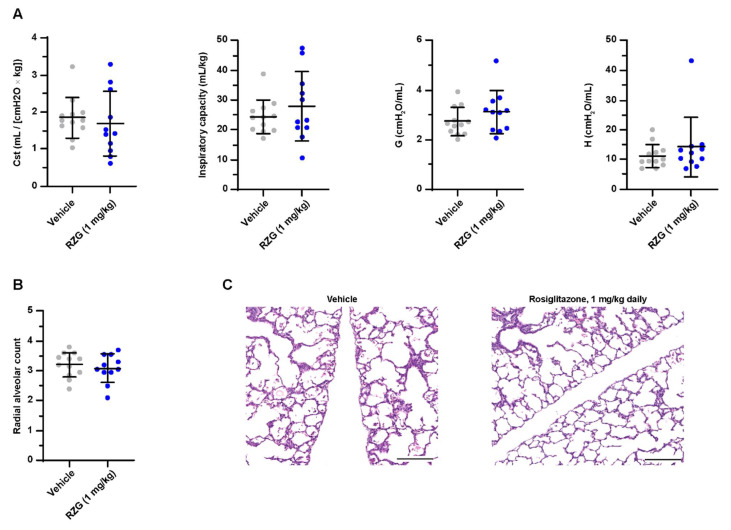
(**A**) Static compliance (C_st_), total inspiratory capacity, lung tissue damping (G), and tissue elastance (H) at post-natal day 7 in preterm rabbits exposed to hyperoxia (95% oxygen) treated with daily intraperitoneal rosiglitazone (1 mg/kg) or the vehicle. (**B**) Radial alveolar count and (**C**) representative hematoxylin–eosin-stained lung sections at post-natal day 7 (the scale bar represents 200 μm). No significant differences were found between groups.

Daily intraperitoneal RGZ doses as low as 0.3 mg/kg significantly promoted surfactant synthesis (i.e., increased lamellar body count and SP-B and SP-C expression) and enhanced alveolarization in newborn rats [23]. Similarly, Dasgupta et al. showed that postnatal RGZ treatment at 1 or 3 mg/kg doses for 7 days significantly improved the RAC and alveolar septal thickness in neonatal rats exposed to hyperoxia (95% O_2_) [10]. In the present study, however, daily postnatal administration of RGZ at 1 mg/kg to preterm rabbits exposed to hyperoxia neither improved lung function (inspiratory capacity, G, H and C_st_) nor alveolarization (RAC, Figure 3). No differences in PND 7 mortality (25% RGZ group vs. 20% vehicle group) or weight gain (43.46 ± 4.84 g RGZ group vs. 46.39 ± 4.99 g vehicle group) were found between groups.

The 1 mg/kg dose was selected based on previous rodent studies showing positive effects on pulmonary outcomes at similar doses [10,23]. Differences in the animal model may partly explain the lack of RGZ efficacy. At 28 days of gestation, preterm rabbits are delivered in the boundaries between the canalicular and saccular phases of lung development, requiring supplemental oxygen for the transition into postnatal life [25]. Conversely, term rodents are born in the middle of the saccular phase, display fully functional lungs, and can breathe room air. Differences in lung maturation at delivery may partly explain the differential responses to hyperoxia between term rodents and preterm rabbits (i.e., the longer exposure time required to induce lung damage in preterm rabbits) [37] and may also explain the different pulmonary response to 1 mg/kg of RGZ.

In a subsequent experimental session, we investigated the pulmonary outcomes of a higher intraperitoneal RGZ dose. To define the RGZ dose, a target engagement (TE%) evaluation was performed based on the unbound plasma and EC50 values for RGZ previously determined in vitro. The free plasma RGZ concentration at each time point of the pharmacokinetic curve was estimated based on the RGZ plasma protein binding (97.5%). TE% at each time point was calculated according to the following formula.

*TE*% = (*cu*/*cu*+ EC50) × 100 (8)

where *cu* is the unbound RGZ and EC50 was 63 nM, as determined in the in vitro PPARγ selectivity assay.

**Figure 4:**
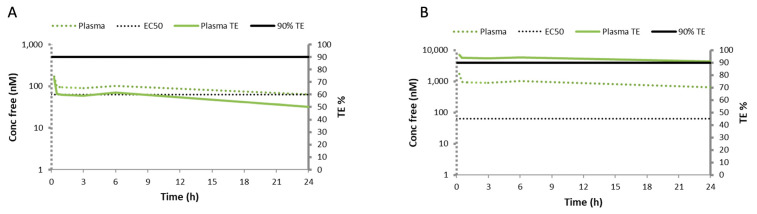
Target engagement percentage (TE%) evaluation based on the pharmacokinetic curve obtained after administering 1 mg/kg of intraperitoneal rosiglitazone and considering the unbound plasma and the EC50 values determined for rosiglitazone in vitro. (**A**) The concentration of free rosiglitazone (Conc free, solid green line), which estimated a target engagement of 50%, far from the optimal 90% TE (solid black line). (**B**) The linear scaling model to an intraperitoneal rosiglitazone dose of 10 mg/kg estimated a robust TE% > 90%, denoted by superimposed plasma and TE% curves.

The TE% evaluation revealed a sub-optimal TE% (40–60%) for the intraperitoneal 1 mg/kg dose (Figure 4A). Since the RGZ plasma levels have been reported to increase in a dose-proportional manner after oral administration in humans [38], a linear scaling model to an intraperitoneal dose of 10 mg/kg was performed, which estimated a robust TE% > 90% (Figure 4B).

**Figure 5:**
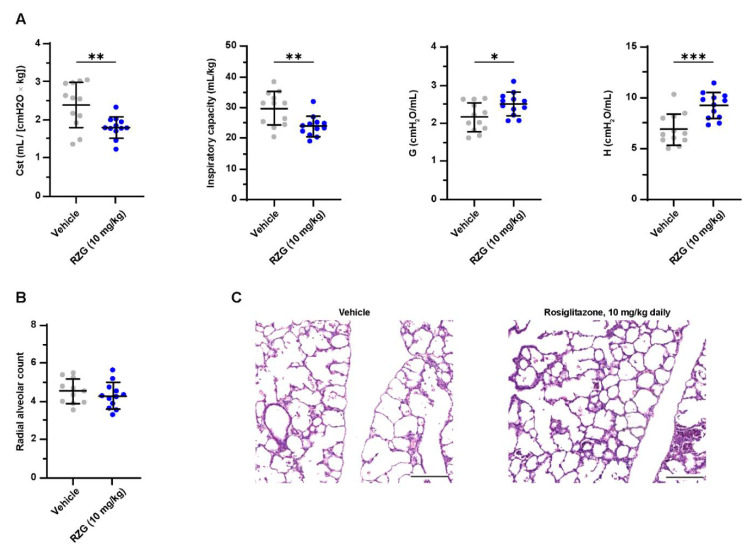
(**A**) Static compliance (C_st_), total inspiratory capacity, lung tissue damping (G), and tissue elastance (H) at post-natal day 7 in preterm rabbits exposed to hyperoxia (95% oxygen) treated with daily intraperitoneal rosiglitazone (10 mg/kg) or vehicle. (**B**) Radial alveolar count and (**C**) representative hematoxylin–eosin-stained lung sections at post-natal day 7 (scale bar: 200 μm). Comparisons between groups were performed using the unpaired, two-sided t-test. * *p* < 0.05; ** *p* < 0.01; and *** *p* < 0.001.

Nevertheless, the daily administration of 10 mg/kg of RGZ had deleterious effects on lung function, showing a significantly lower inspiratory capacity and lung compliance (C_st_) in the pups treated with RGZ compared with the vehicle group (*p* < 0.01, Figure 5). The worsening of lung function was not reflected at the histological level with no difference between groups in the RAC. The negative effect on lung function most probably indicates a toxic effect of the daily 10 mg/kg RGZ dose. However, mortality was similar in the RGZ-treated and vehicle-treated pups. Notably, the plasma of all pups receiving high-dose RGZ had a white, dense appearance, indicative of dyslipidemia. This finding prompted us to investigate the blood lipid levels after daily 1 and 10 mg/kg RGZ treatments.

### 3.5. Daily Rosiglitazone Treatment Induces Dyslipidemia in Premature Rabbits

**Figure 6:**
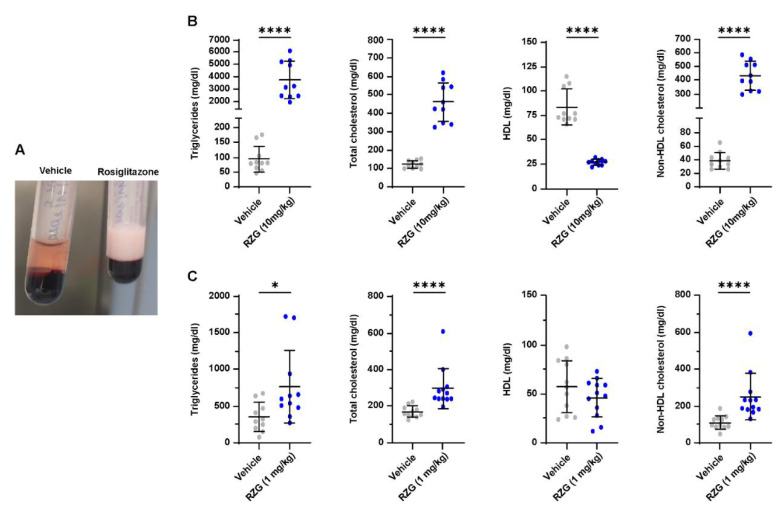
(**A**) A representative image of the macroscopic appearance of the plasma obtained from preterm rabbits exposed to hyperoxia (95% oxygen) for 7 days treated with daily rosiglitazone (RGZ, 10 mg/kg in the example), which shows a white, dense appearance compared with the plasma from another animal receiving the vehicle. (**B**) and (**C**), respectively, show the blood lipid levels in the groups of preterm rabbits exposed to hyperoxia either treated with daily doses of 1 and 10 mg/kg of rosiglitazone or the vehicle. Comparisons between groups were performed using the Mann–Whitney test except the comparisons between RZG 10 mg/kg and the vehicle for total cholesterol and non-HDL cholesterol, which were performed using an unpaired t-test. * *p* < 0.05; and **** *p* < 0.0001.

In the lung, PPARγ activation mediates the uptake of triglycerides from the circulation, promoting the transdifferentiation of fibroblasts into the lipofibroblast phenotype [8]. However, PPARγ activation plays a central role in regulating lipid metabolism and is ubiquitously expressed in several other organs, including the intestine, liver, brain, pancreas, and white and brown adipose tissue [39]. Therefore, even if RGZ administration is intended to improve the pulmonary outcomes in BPD, it may also trigger undesired effects in other organ systems. Wang et al. addressed this issue by determining the blood levels of cholesterol and triglycerides after single (24 h) and repeated (7 days) administration of RGZ to newborn rats. In their study, serum cholesterol and triglycerides levels remained unchanged in the dose range of 0.1–8 mg/kg [23]. Conversely, the present study found an aberrant modulation of serum lipids at PND 7 in RGZ-treated preterm rabbits, particularly in pups treated with the 10 mg/kg dose (*p* < 0.0001 for all lipids, Figure 6). Notably, serum lipid levels were also altered in pups receiving daily 1 mg/kg RGZ, with significantly increased levels of triglycerides (*p* < 0.05), total cholesterol (*p* < 0.01), and non-HDL (*p* < 0.01). To our knowledge, this is the first study reporting RGZ-associated dyslipidemia in premature BPD models. Two previous studies have investigated the use of RGZ in premature rabbits: Richter et al. investigated the antenatal administration of RGZ, showing that RGZ (3 mg/kg) delivered through an intramuscular injection to the mothers 24 and 48 h prior to delivery attenuated the hyperoxic lung injury at PND 7 [17]. Recently, Krishna et al. demonstrated that twice-daily administration of 0.1 mg/kg of RGZ improved myelination and neurological recovery in premature rabbit pups with intraventricular hemorrhage [40]. No issues were raised regarding RGZ-associated dyslipidemia in these studies. However, the systemic exposure to RGZ in these studies could be expected to be low, considering the transplacental administration in the Richter et al. study and the relatively low dose in the Krishna et al. study.

Clinical trials have reported RGZ-associated increases of plasma triglycerides, large- and medium-sized lipoproteins and low- and high-density cholesterol in adult patients with type 2 diabetes and dyslipidemia [33]. Moreover, RGZ administration in diabetic patients increases the risk of heart failure and cardiovascular mortality [41]. Alterations of plasma lipid levels are of particular concern in the most premature babies (<28 weeks’ gestation), who are at the highest risk of developing BPD and are prone to hypertriglyceridemia due to their prematurity-associated low lipoprotein lipase activity and reduced endothelial function [42].

### 3.6. Lung Proteomics Reveal Dysregulation of Inflammatory Pathways and Protein–Lipid Complex by High-Dose Rosiglitazone

The “omics” disciplines, including transcriptomic, metabolomics, and proteomics analyses, may be helpful not only in widening the knowledge regarding the pathogenesis of BPD but also in planning more targeted therapies. Salaets et al. performed a transcriptome analysis with lung homogenates from preterm rabbits to investigate the effects of a 7-day hyperoxia exposure relative to room air management [43]. Hyperoxia exposure dysregulated genes involved in inflammation, reactive oxygen species metabolism, lung development arrest, and vasculogenesis. These findings are in line with the pathophysiology of BPD and validated the preterm rabbit exposed to hyperoxia for 7 days as an experimental model of BPD. Accordingly, Shrestha et al. [44] correlated high-throughput transcriptomic and proteomic analysis on lung samples of a mouse model of BPD, demonstrating that hyperoxia exposure dysregulated the expression of 344 genes and 21 proteins, relentlessly altering those pathways involved in lung development and repair. Takeda et al. performed a lung transcriptome analysis on mice lungs exposed to hyperoxia treated with daily RGZ relative to mice lungs exposed to hyperoxia alone [22]. They found a significant modulation of genes involved in lung development, including muscle differentiation, blood vessel morphogenesis, pattern specification and morphogenesis, and response to stimuli-related genes, suggesting a pro-angiogenic lung response to RGZ treatment.

To the best of our knowledge, the present study is the first study that compared the proteomic profile from lungs obtained from preterm rabbits either treated daily with 1 or 10 mg/kg of RGZ doses or vehicle. We first analyzed the differential proteome profile of the lung of RGZ treatment at 1 mg/kg during hyperoxia relative to hyperoxia alone. In total, 5805 proteins were identified and processed as they met the selected confidence identification criteria. Comparing the RGZ and vehicle groups, 38 and 60 proteins resulted as significantly up and downregulated, respectively (Table S1). However, the Metascape functional enrichment analysis did not identify any significantly modulated pathway after 1 mg/kg RGZ treatment daily administered, according to the cut-off set to assume a pathway as significantly modulated (Log (*q*-value) < −4). The lack of pathway modulation after 1 mg/kg of RGZ suggests that the treatment had little to no effect in the pups’ lungs, although a 1 mg/kg dose sufficed to dysregulate the lipid metabolism in other tissues.

Conversely, the 10 mg/kg dose of RGZ induced a significant modulation of several pathways. In total, 75 and 100 proteins resulted as significantly up and downregulated, respectively (Table S2). Metascape functional enrichment analysis of these proteins showed upregulation of the inflammatory pathways, such as neutrophil degranulation and regulation of the inflammatory response. Moreover, upregulation of the protein–lipid complex remodeling was also detected and directly related to the overexpression of apolipoprotein isoforms (Table 3).

On the contrary, the downregulation of processes involved in DNA replication initiation and elongation, and extracellular matrix (ECM) organization were also determined. Here, the negative modulation occurred on proteins involved in binding and recognition of the structural component of the ECM and on COL4A6, the unique collagen isoform modulated out of 11 isoforms identified, whereas differential expression was measured for any matrix-degradative metalloproteinase family (Table S2). A similar level of surfactant protein expression indicated that the treatment with 10 mg/kg RGZ did not affect the pulmonary surfactant synthesis and secretion. Therefore, the outcomes of the proteomic analysis indicate deleterious effects of daily RGZ administration at 10 mg/kg, denoted by the dysregulation of the ECM organization pathways and the upregulation of lung inflammation and lipid metabolism pathways, which may explain the adverse functional respiratory outcomes observed at PND 7.

## 4. Limitations of the Study

The present study has some limitations. First, we did not include normoxia control groups (21% O_2_) in the experimental design to reduce the number of animals. This decision was taken based on the well-characterized hyperoxic injury in the preterm rabbit, which we, and others, have validated in several studies [26,27,28,29,45]. Here, we report the pulmonary outcomes of just two doses of RGZ at 1 and 10 mg/kg, respectively, showing no effects and worsening lung function. Therefore, it remains unknown if other TZDs or intermediate doses of RGZ could improve lung function in this model. However, it should be considered that daily 1 mg/kg RGZ doses already produced a significant increase in blood lipids, which could also be expected for intermediate RGZ doses. Future studies in preterm rabbits are warranted to investigate the efficacy of other TZDs, such as PGZ, which has shown beneficial effects in rodent models of BPD [19,21] and has been shown to have significantly different effects on plasma lipids compared with RZG in type II diabetes patients (i.e., a significant reduction in triglycerides) [46].

## 5. Conclusions

The three TZDs investigated here, RGZ, PGZ, and DRF-2546, showed high epithelial permeability and plasma and lung tissue binding in vitro, although RGZ showed the highest affinity for PPARγ. The pharmacokinetic profiling of RZG in preterm rabbits revealed an equivalent biodistribution after either intratracheal or intraperitoneal administration.

Daily intraperitoneal RGZ doses of 1 mg/kg did not improve lung function or the RAC in preterm rabbits exposed to prolonged hyperoxia. Moreover, increasing the RGZ dose to 10 mg/kg caused a significant lung function worsening, which was associated with the upregulation of the lung inflammation and lipid metabolism pathways and downregulation of the ECM organization. Notably, daily postnatal RGZ dosing caused an aberrant modulation of serum lipids, particularly in rabbit pups treated with the 10 mg/kg dose.

## Figures and Tables

**Table 1:** Plasma protein binding, lung tissue binding, and apparent permeability through Caco-2 cell monolayers of thiazolidinediones.

TZD Type	Plasma Protein Binding (%)	Lung Tissue Binding (%)	*P_app_* (A–B/B–A, cm/sec)	*P_app_* with *P_gp_* Inhibitor (A–B/B–A, cm/sec)
RGZ	97.5 ± 0.2	94.2 ± 0.7	1.45 × 10^−5^ ± 0.06 × 10^−5^ / 1.00 × 10^−5^ ± 0.06 × 10^−5^	1.63 × 10^−5^ ± 0.05 × 10^−5^ / 1.14 ×10^−5^ ± 0.04 × 10^−5^
PGZ	96.7 ± 0.6	95.6 ± 2.1	1.18 × 10^−5^ ± 0.05 × 10^−5^ / 1.53 × 10^−5^ ± 0.36 × 10^−5^	1.23 × 10^−5^ ± 0.17 × 10^−5^ / 2.54 × 10^−5^ ± 0.55 × 10^−5^
DRF-2546	98.4 ± 0.3	91.8 ± 1.8	2.29 × 10^−5^ ± 0.03 × 10^−5^ / 1.87 × 10^−5^ ± 0.80 × 10^−5^	2.86 × 10^−5^ ± 0.15× 10^−5^ / 1.58 × 10^−5^ ± 0.34 × 10^−5^

*P_app_*, apparent permeability; *P_gp_*, P-glycoprotein transporter; A–B, apical-to-basolateral transport; B–A, basolateral-to-apical transport; TZD, thiazolidinediones; RGZ, rosiglitazone; PGZ, pioglitazone.

**Table 2:** Pharmacokinetic parameters after either intratracheal or intraperitoneal administration of rosiglitazone (1 mg/kg) to premature rabbit pups.

	T_max_ (h)	C_max_ (ng/mL)	t_1/2_ (h)	AUC_last_ (ng/mL∙h)	AUC_Lung_/AUC_Plasma_
**Intratracheal Administration**					
**Lung**	0.25	1067	15	17,020	0.7
**Plasma**	0.25	1950	15	25,785	
**Intraperitoneal Administration**					
**Lung**	0.25	1024	n.c.	19,752	0.7
**Plasma**	0.25	2443	n.c.	29,490	

T_max_, time of the maximum concentration; C_max_, maximum concentration; t_1/2_, half-life (calculable only in case of well-defined elimination phase); AUC_last_, area under the curve up to the last time point; AUC_Lung_/AUC_Plasma_, lung to plasma ratio of the AUC_last_ of each compartment; n.c., not calculable.

**Table 3:** Selected pathways significantly modulated by daily RGZ administration of 10 mg/kg.

Pathway Description	Downregulated Log (*q*-Value)	Upregulated Log (*q*-Value)	Dysregulated Proteins in the Pathway ***
1.Neutrophil degranulation	-	−12.0	AHSG, CAMP, CHIT1, FTL, HK3, HP, ITGAM, LCN2, LTF, MPO, CFP, S100A8, S100A9, S100A12, PGLYRP1, SNAP29, GCA, PYCARD, RETN
2. MCM * complex	−7.6	-	MCM2, MCM3, MCM4, MCM5, MCM7
3. Regulation of inflammatory response	-	−7.1	AGT, AHSG, APOE, IL16, LBP, MVK, S100A8, S100A9, S100A12, SNCA, PGLYRP1, PYCARD, PGLYRP2
4. Humoral immune response	-	−7.0	C6, C8A, C8B, C8G, CAMP, CRP, HPX, LTF, CFP, S100A9, S100A12, PGLYRP1
5. Transition metal ion homeostasis		−5.4	FTL, HPX, LCN2, LTF, S100A8, S100A9, ABCB6, STEAP4
6. Positive regulation of reactive oxygen species metabolic process	-	−4.7	AGT, CRP, ITGAM, LCN2, SNCA, XDH
7. Protein–lipid complex remodeling	-	−5.0	AGT, APOB, APOC3, APOE, MPO
8. Regulation of endopeptidase activity		−5.0	AGT, AHSG, BAX, LTF, S100A8, S100A9, SNCA, XDH, LAMTOR5, FETUB, PYCARD
9. Extracellular matrix organization	−5.1		COL4A6, HAPLN1, DCN, FBLN2, FBN2, ICAM2, ITGA6, ITGB3, SPARC, PXDN, CRTAP
10. NABA CORE MATRISOME **	−4.6		COL4A6, HAPLN1, DCN, FBLN2, FBN2, SPARC, PXDN, SPARCL1, POSTN, NPNT

* MCM: minichromosome maintenance protein complex. ** NABA CORE MATRISOME: Ensemble of genes encoding core extracellular matrix including extracellular matrix glycoproteins, collagens and proteoglycans. *** Extended protein names available in the Table S2.

## Data Availability

Not applicable.

## Supplementary Materials

The following supporting information can be downloaded at: https://www.mdpi.com/article/10.3390/pharmaceutics14071507/s1, Table S1: List of proteins significantly modulated by the treatment with rosiglitazone 1 mg/kg. Proteins were considered significantly upregulated in case of Log (*p*-value) ≥ 1.3 and Log_2_ (Fold-change) ≥ 0.5849 and significantly downregulated in case of Log(*p*-value) ≥ 1.3 and Log_2_ (Fold-change) ≤ −0.5849; Table S2: List of proteins significantly modulated by the treatment with rosiglitazone 10 mg/kg. Proteins were considered significantly upregulated in case of Log(p-value) ≥ 1.3 and Log_2_ (Fold-change) ≥ 0.5849 and significantly downregulated in case of Log(p-value) ≥ 1.3 and Log_2_ (Fold-change) ≤ −0.5849.
