# Supplementary material for: Daily Intraperitoneal Administration of Rosiglitazone Does Not Improve Lung Function or Alveolarization in Preterm Rabbits Exposed to Hyperoxia
Source: Pharmaceutics. 2022 Jul 20;14(7):1507. doi: 10.3390/pharmaceutics14071507 (PMC9320886; doi:10.3390/pharmaceutics14071507)
Supplement: Supplementary file 1 [file pharmaceutics-14-01507-s001.zip › pharmaceutics-1792563-supplementary.pdf]

Supplemental data

Daily Intraperitoneal Administration of Rosiglitazone Does Not Improve Lung Function or Alveolarization in Preterm Rabbits Exposed to Hyperoxia

Table S1. List of proteins significantly modulated by the treatment with rosiglitazone 1 mg/kg. Proteins were considered significantly upregulated in case of Log (*p*-value) ≥ 1.3 and Log_2 (Fold-change) ≥ 0.5849 and significantly downregulated in case of Log(*p*-value) ≥ 1.3 and Log_2 (Fold-change) ≤ −0.5849.

Protein Description	Genename	−Log (*p*-value)	Log2 (FC)
Myelin P2 protein	PMP2	4.71	2.44
Mitochondrial ribosomal protein L24	MRPL24	1.30	1.73
ROS proto-oncogene 1, receptor tyrosine kinase	ROS1	1.42	1.53
Tyrosine-protein kinase	FYN	1.44	1.44
Collectin subfamily member 12	COLEC12	1.80	1.13
Amine oxidase	MAOA	1.94	1.09
Sideroflexin 3	SFXN3	1.58	1.08
PHD finger protein 23	PHF23	2.96	1.07
Matrilin 2	MATN2	1.80	1.05
Histone deacetylase 3	HDAC3	2.47	0.98
Spectrin beta chain	SPTB	3.02	0.98
Adducin 2	ADD2	1.90	0.98
MAP7 domain-containing protein 3	MAP7D3	2.31	0.96
RNA binding motif protein 5	RBM5	1.83	0.92
CD59 glycoprotein	CD59	1.62	0.90
Fraser extracellular matrix complex subunit 1	FRAS1	1.31	0.86
Synuclein alpha	SNCA	1.32	0.86
Calcitonin receptor like receptor	CALCRL	1.86	0.80
Somatomedin B and thrombospondin type 1 domain containing	SBSPON	2.17	0.79
Adenylate kinase isoenzyme 1	AK1	2.50	0.78
Syndecan 4	SDC4	1.46	0.77
Proline and arginine rich end leucine rich repeat protein	PRELP	1.90	0.73
Myristoylated alanine rich protein kinase C substrate	MARCKS	1.56	0.72
Carbonic anhydrase 4	CA4	2.24	0.72
Spindlin 1	SPIN1	1.55	0.71
Periaxin	PRX	1.30	0.67
TBC1 domain family member 5	TBC1D5	1.65	0.66
Annexin	ANXA4	1.57	0.65
Family with sequence similarity 192 member A	FAM192A	1.87	0.65
Prohibitin	PHB	2.26	0.64
Dipeptidase	DPEP1	2.21	0.63
Retinol-binding protein 4	RBP4	1.69	0.63
WD repeat domain 3	WDR3	1.50	0.62
MICOS complex subunit	CHCHD3	1.38	0.61
Apolipoprotein C-I	apoCI	2.20	0.60
Thymidine kinase 2, mitochondrial	TK2	1.62	0.60
Collagen alpha-2(I) chain	COL1A2	1.40	0.59
Mitochondrial ribosomal protein S26	MRPS26	1.52	0.59
Proteasome assembly chaperone 4	PSMG4	1.91	-0.59

Armadillo like helical domain containing 3	ARMH3	1.60	-0.60
Signal peptide peptidase like 2A	SPPL2A	1.67	-0.60
Bactericidal permeability-increasing protein	BPI	1.43	-0.61
Dedicator of cytokinesis 8	DOCK8	1.86	-0.61
UBIQUITIN_CONJUGAT_2 domain-containing protein	UBE2I	1.55	-0.62
Eukaryotic translation initiation factor 4E family member 2	EIF4E2	1.83	-0.62
Antioxidant 1 copper chaperone	ATOX1	1.77	-0.62
Granulin precursor	GRN	1.47	-0.63
4a-hydroxytetrahydrobiopterin dehydratase	PCBD2	1.40	-0.63
Exosome component 10	EXOSC10	1.40	-0.63
Deoxyhypusine synthase	DHPS	1.81	-0.63
Cilia and flagella associated protein 36	CFAP36	1.76	-0.64
WD repeat domain 54	WDR54	2.21	-0.64
Myeloperoxidase	MPO	1.66	-0.64
Phospholysine phosphohistidine inorganic pyrophosphate phosphatase	LHPP	1.85	-0.64
Phospholipase B-like	PLBD2	1.84	-0.67
Mitochondrial ribosomal protein L44	MRPL44	1.57	-0.69
Formin binding protein 4	FNBP4	1.76	-0.69
Cytosolic phospholipase A2	PLA2G4A	1.72	-0.69
Dystonin	DST	1.43	-0.70
Cathepsin A	CTSA	1.93	-0.70
Threonine synthase like 2	THNSL2	1.31	-0.71
Karyopherin subunit alpha 1	KPNA1	1.73	-0.72
Galectin-9	LGALS9	1.61	-0.75
Microtubule-associated proteins 1A/1B light chain 3C	MAP1LC3C	2.30	-0.76
CD109 molecule	CD109	2.10	-0.76
Golgi phosphoprotein 3 like	GOLPH3L	1.38	-0.78
STEAP4 metalloreductase	STEAP4	1.43	-0.79
Tetraspanin	CD82	1.42	-0.80
Asparagine synthetase [glutamine-hydrolyzing]	ASNS	2.24	-0.81
Interferon induced protein with tetratricopeptide repeats 3	IFIT3	1.93	-0.81
Colony stimulating factor 1 receptor	CSF1R	2.75	-0.85
V-set and immunoglobulin domain containing 4	VSIG4	2.29	-0.86
Pentraxin 3	PTX3	1.88	-0.86
Mitochondrial ribosomal protein S9	MRPS9	1.38	-0.87
TP53 induced glycolysis regulatory phosphatase	TIGAR	1.48	-0.87
Myotubularin related protein 14	MTMR14	1.56	-0.88
Cathepsin K	CTSK	2.54	-0.89
WD repeat domain 11	WDR11	1.42	-0.90
Metalloproteinase inhibitor 1	TIMP1	1.44	-0.91
Immunoglobulin J chain	JCHAIN	1.95	-0.92
Ribosome production factor 2 homolog	RPF2	1.36	-0.92
Phospholipid transfer protein	PLTP	1.71	-0.93
Tetraspanin	TSPAN1	1.84	-0.93
Leucine rich pentatricopeptide repeat containing	LRPPRC	1.78	-0.95
Bystin like	BYSL	1.80	-0.96
2'-5'-oligoadenylate synthetase 3	OAS3	1.66	-1.01
Vav guanine nucleotide exchange factor 1	VAV1	2.39	-1.02
EMG1 N1-specific pseudouridine methyltransferase	EMG1	1.64	-1.03
DDB1- and CUL4-associated factor 11	DCAF11	2.19	-1.05

Guanylate binding protein 1	GBP1	1.41	-1.06
Negative elongation factor complex member C/D	NELFCD	2.56	-1.13
Tyrosyl-tRNA synthetase 2	YARS2	2.14	-1.20
Uncharacterized protein	AKR1C1L	1.73	-1.20
2'-5'-oligoadenylate synthetase 2	OAS2	1.60	-1.24
Malonyl-CoA-acyl carrier protein transacylase	MCAT	2.56	-1.38
Hcy-binding domain-containing protein	BHMT	1.35	-1.46
Fructose-bisphosphate aldolase B	ALDOB	1.57	-1.48
Arginase	ARG1	4.16	-1.89

Table S2. List of proteins significantly modulated by the treatment with rosiglitazone 10 mg/kg. Proteins were considered significantly upregulated in case of Log(p-value) ≥ 1.3 and Log_2 (Fold-change) ≥ 0.5849 and significantly downregulated in case of Log(p-value) ≥ 1.3 and Log_2 (Fold-change) ≤ -0.5849.

Protein Description	Genename	-Log(*p*-value)	Log2(FC)
C-reactive protein	CRP	1.54	2.51
STEAP4 metalloreductase	STEAP4	1.76	2.05
Myelin P2 protein	PMP2	5.44	1.97
Histone deacetylase 3	HDAC3	5.13	1.85
WASP actin nucleation promoting factor	WAS	3.62	1.81
Late endosomal/lysosomal adaptor, MAPK and MTOR activator 5	LAMTOR5	1.42	1.67
Complement component C8 alpha chain	C8A	2.90	1.59
Apolipoprotein C-III	APOC3	3.04	1.58
Peptidoglycan recognition protein 2	PGLYRP2	4.24	1.54
ABI family member 3	ABI3	1.41	1.53
Resistin	RETN	3.36	1.49
C-C motif chemokine ligand 14	CCL14	1.72	1.47
Protein S100-A12	S100A12	2.22	1.39
Aldehyde oxidase 4	AOX4	1.74	1.39
BCL2 associated X, apoptosis regulator	BAX	2.90	1.38
Protein S100	S100A8	2.30	1.37
Sequestosome 1	SQSTM1	2.77	1.37
Lactotransferrin	LTF	2.19	1.34
BolA-like protein 3	BOLA3	2.16	1.33
Spectrin beta chain	SPTB	2.62	1.25
Complement component C8 gamma chain	C8G	2.28	1.24
Syndecan 4	SDC4	2.16	1.22
Protein S100	S100A9	2.62	1.21
Lipocalin 2	LCN2	2.00	1.21
Apolipoprotein B	APOB	2.50	1.16
Ubiquitin specific peptidase 19	USP19	1.34	1.16
Lipopolysaccharide-binding protein	LBP	1.46	1.13
Mevalonate kinase	MVK	2.39	1.13
Ribosomal RNA processing 15 homolog	RRP15	1.58	1.12
G protein-coupled receptor class C group 5 member C	GPRC5C	2.56	1.10
Peptidoglycan-recognition protein	PGLYRP1	2.78	1.10
X-prolyl aminopeptidase 2	XPNPEP2	1.91	1.07
RNA binding motif protein 19	RBM19	1.33	1.05
Grancalcin	GCA	3.08	1.05
Hexokinase 3	HK3	4.15	1.04
Macrophage stimulating 1	MST1	2.90	1.04
PYD and CARD domain containing	PYCARD	1.48	1.01
Chromosome 11 open reading frame 96	C11orf96	1.45	1.00
Coagulation factor V	F5	1.67	0.98
Chitinase 1	CHIT1	1.62	0.97
Haptoglobin	HP	3.29	0.94
Solute carrier family 4 member 1 (Diego blood group)	SLC4A1	3.01	0.92
CAP18_C domain-containing protein	CAMP	2.71	0.91
Ferritin light chain	FTL	2.11	0.88
ATP binding cassette subfamily B member 6 (Langereis blood group)	ABCB6	2.19	0.87
Chromosome 9 open reading frame 78	C9orf78	2.28	0.87

Myeloperoxidase	MPO	1.70	0.85
Fetuin B	FETUB	3.29	0.84
Ras-related protein Rab-32	RAB32	2.50	0.80
Integrin subunit alpha M	ITGAM	1.71	0.79
4-hydroxyphenylpyruvate dioxygenase	HPD	1.42	0.78
Angiotensinogen	AGT	5.10	0.78
Synapse associated protein 1	SYAP1	1.99	0.77
Apolipoprotein C-I	apoCI	2.51	0.77
Pre-mRNA-splicing factor 18	PRPF18	1.34	0.75
Haloacid dehalogenase like hydrolase domain containing 3	HDHD3	2.10	0.75
Microtubule interacting and trafficking domain containing 1	MITD1	2.61	0.73
Apolipoprotein E	APOE	2.00	0.73
Solute carrier family 25 member 1	SLC25A1	1.42	0.71
Multiple coagulation factor deficiency protein 2	MCFD2	1.46	0.68
Synuclein alpha	SNCA	1.51	0.66
Ubiquitin associated protein 2	UBAP2	1.34	0.64
Complement C6	C6	2.66	0.64
Alpha-2-HS-glycoprotein	AHSG	2.84	0.64
Hemopexin	HPX	1.33	0.64
Complement component C8 beta chain	C8B	3.29	0.64
Pro-interleukin-16	IL16	1.47	0.63
WAPL cohesin release factor	WAPL	1.86	0.63
Complement factor properdin	CFP	1.88	0.62
Coiled-coil domain containing 12	CCDC12	1.36	0.61
Vitamin D-binding protein	GC	4.36	0.61
4HBT domain-containing protein	ACOT13	2.78	0.61
Integrator complex subunit 12	INTS12	1.43	0.59
t-SNARE coiled-coil homology domain-containing protein	SNAP29	1.52	0.59
WD repeat domain 43	WDR43	1.54	0.59
Roundabout guidance receptor 4	ROBO4	1.44	-0.59
Transforming growth factor beta-1-induced transcript 1 protein	TGFB1I1	3.77	-0.59
IF rod domain-containing protein	KRT18	2.18	-0.59
Peroxidasin	PXDN	3.43	-0.59
Uveal autoantigen with coiled-coil domains and ankyrin repeats	UACA	2.19	-0.59
Fibronectin leucine rich transmembrane protein 3	FLRT3	1.67	-0.59
Proliferating cell nuclear antigen	PCNA	3.82	-0.60
Protein tyrosine phosphatase 4A2	PTP4A2	3.05	-0.60
Calcium binding protein 39 like	CAB39L	1.60	-0.60
Intercellular adhesion molecule 2	ICAM2	1.82	-0.60
TATA box-binding protein-like 1	TBPL1	1.77	-0.62
Ras-related protein Rab-25	RAB25	1.35	-0.63
Minichromosome maintenance complex component 5	MCM5	1.89	-0.63
Integrin subunit alpha 6	ITGA6	2.27	-0.63
RAB3A interacting protein	RAB3IP	1.54	-0.63
Solute carrier family 27 member 3	SLC27A3	1.32	-0.64
Eukaryotic elongation factor 2 kinase	EEF2K	1.91	-0.64
ATP-dependent translocase ABCB1	ABCB1	1.61	-0.65
Ribosomal protein L37	RPL37	1.92	-0.65
SPARC	SPARC	2.30	-0.65
Ectonucleoside triphosphate diphosphohydrolase 1	ENTPD1	3.58	-0.65
Intraflagellar transport protein 27 homolog	IFT27	1.51	-0.65

Vimentin	VIM	1.60	-0.65
Neuronal cell adhesion molecule	NRCAM	1.42	-0.66
Prolylcarboxypeptidase	PRCP	2.13	-0.66
Vesicle amine transport 1 like	VAT1L	2.93	-0.66
Tetratricopeptide repeat domain 9C	TTC9C	1.64	-0.67
Cartilage associated protein	CRTAP	2.26	-0.68
Docking protein 1	DOK1	2.55	-0.68
Filamin binding LIM protein 1	FBLIM1	2.10	-0.69
Nuclear factor I B	NFIB	2.54	-0.69
Glycoprotein nmb	GPNMB	1.60	-0.69
Fibrillin 2	FBN2	3.22	-0.69
G protein-coupled receptor kinase	GRK6	1.55	-0.71
Cyclin K	CCNK	1.50	-0.72
Platelet-derived growth factor receptor beta	PDGFRB	3.34	-0.72
CD300 molecule like family member g	CD300LG	1.32	-0.72
GIT ArfGAP 1	GIT1	1.73	-0.73
Fibulin 2	FBLN2	2.22	-0.73
DNA replication licensing factor MCM7	MCM7	1.79	-0.73
Cyclin dependent kinase 7	CDK7	1.41	-0.74
Microtubule associated protein RP/EB family member 3	MAPRE3	1.30	-0.75
Phosphate cytidylyltransferase 1, choline, alpha	PCYT1A	4.77	-0.75
Stathmin	STMN1	2.55	-0.76
DNA helicase	LPH	2.96	-0.76
Signal transducing adaptor molecule 2	STAM2	2.04	-0.76
STT3 oligosaccharyltransferase complex catalytic subunit B	STT3B	1.57	-0.76
Amyloid beta precursor like protein 2	APLP2	1.57	-0.78
Apoptotic peptidase activating factor 1	APAF1	1.39	-0.78
Acyl-CoA thioesterase 7	ACOT7	2.11	-0.78
SEC11 homolog A, signal peptidase complex subunit	SEC11A	1.36	-0.79
Rabenosyn, RAB effector	RBSN	1.63	-0.81
Dedicator of cytokinesis 4	DOCK4	1.80	-0.81
Negative regulator of ubiquitin like proteins 1	NUB1	1.45	-0.83
CD93 molecule	CD93	2.34	-0.84
Ectonucleotide pyrophosphatase/phosphodiesterase 4	ENPP4	1.56	-0.84
Minichromosome maintenance complex component 2	MCM2	3.58	-0.86
Protein disulfide isomerase family A member 5	PDIA5	1.97	-0.88
Calmodulin regulated spectrin associated protein family member 2	CAMSAP2	1.91	-0.90
Sperm associated antigen 1	SPAG1	1.43	-0.91
Nephronectin	NPNT	1.62	-0.91
Protein arginine methyltransferase 3	PRMT3	1.40	-0.92
Beta-carotene oxygenase 2	BCO2	1.50	-0.92
SWI/SNF related, matrix associated, actin dependent regulator of chromatin subfamily c member 1	SMARCC1	2.07	-0.93
Immunoglobulin superfamily containing leucine rich repeat	ISLR	1.51	-0.94
DNA helicase	MCM4	3.48	-0.94
Phosphate regulating endopeptidase homolog X-linked	PHEX	1.79	-0.94
ARVCF delta catenin family member	ARVCF	1.82	-0.94
Periostin	POSTN	3.16	-0.97
Rho guanine nucleotide exchange factor 26	ARHGEF26	1.65	-0.97
Metalloproteinase inhibitor 3 (Fragment)	TIMP3	2.02	-0.98

Integrator complex subunit 2	INTS2	1.75	-0.99
DAB adaptor protein 2	DAB2	1.59	-1.01
FSHD region gene 1	FRG1	2.01	-1.03
Protein pelota homolog	PELO	1.56	-1.04
Chromosome X open reading frame 38	CXorf38	1.39	-1.06
Collagen type IV alpha 6 chain	COL4A6	1.31	-1.08
KN motif and ankyrin repeat domains 1	KANK1	2.70	-1.14
Ribonuclease 4	RNASE4	2.49	-1.15
Protein kinase domain-containing protein	NEK7	1.42	-1.18
Decorin	DCN	1.85	-1.18
DNA helicase	MCM3	3.03	-1.20
Euchromatic histone lysine methyltransferase 1	EHMT1	1.96	-1.21
acidPPc domain-containing protein	PLPP3	2.19	-1.25
Calcitonin receptor like receptor	CALCRL	1.86	-1.26
TPD52 like 1	TPD52L1	2.30	-1.27
Par-3 family cell polarity regulator beta	PARD3B	2.53	-1.29
SPARC like 1	SPARCL1	2.25	-1.31
5-demethoxyubiquinone hydroxylase, mitochondrial	COQ7	1.58	-1.35
Integrin beta-3	ITGB3	2.04	-1.39
E1A binding protein p300	EP300	2.59	-1.46
AKT interacting protein	AKTIP	2.21	-1.46
TRPM8 channel associated factor 1	TCAF1	1.40	-1.46
PDZ and LIM domain protein 4	PDLIM4	2.38	-1.47
Ribonucleotide reductase regulatory subunit M2	RRM2	3.14	-1.49
Collectin subfamily member 12	COLEC12	2.24	-1.58
Death associated protein kinase 1	DAPK1	1.77	-1.97
Hyaluronan and proteoglycan link protein 1	HAPLN1	1.94	-2.27
Nucleoporin 210	NUP210	1.54	-2.34
Chromosome 2 open reading frame 49	C2orf49	1.70	-3.13
